# RPS6KA5 methylation predict response to 6-week treatment for adolescent MDD patients

**DOI:** 10.1186/s12888-022-04196-4

**Published:** 2022-08-19

**Authors:** Peiwei Xu, Yuanmei Tao, Hang Zhang, Meijiang Jin, Hanmei Xu, Shoukang Zou, Fang Deng, Lijuan Huang, Hong Zhang, Xiaolan Wang, Xiaowei Tang, Zaiquan Dong, Yanping Wang, Li Yin, Xueli Sun

**Affiliations:** 1 Department of Psychiatry, West China Hospital of Sichuan University, No. 28 Dianxin South Street, Chengdu, 610041 Sichuan China; 2 Frontier Science Center for Disease-Related Molecular Networks, Chengdu, 610041 Sichuan China; 3 Sichuan Clinical Medical Research Center for Mental Disorders, Chengdu, 610041 Sichuan China

**Keywords:** Major depressive disorder, Adolescent, Chronic childhood stress, DNA methylation, Treatment response

## Abstract

**Objective:**

We aimed to investigate the effect of differentially methylated genes and chronic childhood stress on the development of depressive symptoms in Chinese adolescents, as well as to test whether methylation at baseline can be used as a predictor of remission at follow-up after six weeks of treatment.

**Methods:**

After recruiting 87 MDD patients and 53 healthy controls, we compared demographic and baseline clinical characteristics. The Childhood Chronic Stress Questionnaire was used to assess stress caused by early-life events. MDD patients underwent six weeks of treatment, and response to treatment was assessed using the Beck Depression Inventory-II. In addition, four MDD patients and five controls were randomly chosen for genome-wide methylation analysis.

**Results:**

The gene RPS6KA5 showed significant methylation differences between the two groups. Severity of chronic childhood stress was significantly associated with increased risk of depression in adolescents, but not with treatment response. Baseline RPS6KA5 methylation can predict remission after six weeks of treatment. We did not observe any interaction between RPS6KA5 methylation and chronic childhood stress.

**Conclusions:**

Our results suggest that RPS6KA5 methylation can be used as a predictor of response to treatment in adolescent MDD patients. Here we offer new evidence for the role of epigenetics in early response to treatment of depression.

**Trial registration:**

ChiCTR, ChiCTR2000033402, 31/05/2020, http://www.chictr.org.cn/index.aspx

**Supplementary Information:**

The online version contains supplementary material available at 10.1186/s12888-022-04196-4.

## Introduction

Major depressive disorder (MDD) is a prevalent chronic mental disorder characterized by depressed mood or loss of interest or pleasure [1]. MDD can cause significant suffering and affect the well-being of adolescents: in China, MDD affects 1.6% of middle school students and 2.8% of high school students [2]. In addition to innate genetic factors, childhood adversity such as abuse, neglect, and bullying (defined here as exposure to stressful or unsuitable environmental conditions) can increase an individual’s vulnerability to depression [3]. Epigenetics, particularly DNA methylation, has been proposed as one of the major mechanisms involved in this crosstalk [4]: for example, early life events can influence gene expression through modifications in DNA methylation and increase susceptibility to depression [5–7].

Methylation refers to the regulation of gene expression by a family of DNA methyltransferases via the addition of a methyl group to the 5-carbon of the cytosine base. Studies have demonstrated that the abnormal methylation of genes can result in disease progression, and that relatively stable methylation patterns can act as biomarkers for the diagnosis and treatment of depression [8, 9]. These studies focused on genes that had previously been implicated in the pathophysiology of depressive disorder and found to be frequently methylated.

Currently, the well-established monoamine hypothesis aims to explain depressive phenotypes based on an imbalance in monoamines such as serotonin and norepinephrine [10]. A majority of antidepressants exert their effects by affecting levels or activity of monoamine neurotransmitters [11]. However, monoaminergic neurotransmission is a complicated process, downstream changes in signaling pathways related to inflammation, immunity, neural plasticity and neurotrophic factors, may serve a critical role [12, 13]. The monoamine hypothesis, along with the neurotrophic and hypothalamic-pituitary axis dysfunction hypotheses, associate the methylation of BDNF, SLC6A4, and NR3C1 with MDD [14, 15]. Other work has suggested that the methylation of BDNF, SLC6A4, HTR1A, HTR1B, IL11, and potentially other genomic loci may help predict clinical response [16]. For example, small sample studies involving Chinese patients have confirmed the ability of HTR1A/1B and BDNF methylation to predict the response to antidepressant therapy [17, 18].

Compared to candidate-gene studies, genome-wide DNA methylation analyses may offer a less biased, data-driven approach that can identify novel genes with potential value for clinical practice. However, the findings of such analyses remain inconsistent [14]. In a Swedish population-based cohort study of adolescents, methylation at an miR-4646-associated locus correlated with risk of developing MDD [19]. Further exploratory studies are required to identify new candidate genes associated with depression, as well as to examine how specific environmental stressors exacerbate the disorder. Therefore, in this study, we aimed to identify novel genetic markers for the early detection of MDD in adolescents and for prediction of response to therapy. We performed second-generation high-throughput sequencing and bioinformatic analyses to screen and analyze differentially methylated genes in Chinese patients, and we focused on the differentially methylated gene RPS6KA5 because it is involved in the neurotrophic factor cascades and is abundantly expressed in the human brain. We hypothesized that methylation of this gene, perhaps influenced by chronic childhood stress, would be associated with MDD severity and treatment response.

## Methods

### Study subjects

We recruited 87 first-episode, drug-naive adolescent MDD patients with a baseline score of ≥ 20 on the Chinese version of Beck Depression Inventory-II (BDI) [20] from the Department of Psychiatry at West China Hospital of Sichuan University. All recruited patients were independently diagnosed by two senior psychiatrists using the Chinese version of the Kiddie Schedule for Affective Disorders and Schizophrenia-Present and Lifetime Version [21], as well as the guidelines recommended in the Diagnostic and Statistical Manual of Mental Disorders (4th edition) [22]. Additionally, we used posters to recruit 53 healthy adolescents from the general community to serve as controls. All healthy controls (HC) underwent neurological examinations and detailed semi-structured interviews to ensure that they had a baseline BDI score < 20 and that they had no history of psychiatric disease or suicide attempts.

We included MDD patients and controls between the ages of 12 and 17 years. All participants had to have completed at least elementary schooling in order to ensure that they would understand the content of the psychological scales. We excluded adolescents with a history of electroconvulsive treatment, physical disease, psychotropic drug use, and alcohol or drug abuse, as well as those suffering from severe physical illness or other axis I/II mental illnesses. This study was approved by the Ethics Committee of the West China Hospital of Sichuan University. Written informed consent was provided by all participants and their guardians.

### Assessment of childhood chronic stress

We used the Childhood Chronic Stress Questionnaire (CCSQ) to evaluate baseline levels of chronic stress in all participants. The CCSQ is a 60-item retrospective self-report instrument for children and adolescents that includes questions based on three major dimensions: peer bullying (15 items), childhood abuse and neglect (29 items), and adverse childhood experiences (16 items). The CCSQ is evaluated on a 6-point scale, and has good validity and reliability. This questionnaire has been validated for use in the Chinese population [23].

### Treatment and primary outcome

The MDD patients underwent an open-label antidepressant trial for six weeks, and their daily doses were adjusted by their clinicians based on routine clinical practice. Among these patients, 35 received sertraline (25–150 mg/d), 18 received agomelatine (50 mg/d), 17 received escitalopram (10–20 mg/d), two received fluoxetine (20–40 mg/d), three received venlafaxine (150–225 mg/d), one received duloxetine (120 mg/d), one received paroxetine (25 mg/d), one received sertraline (100 mg/d) and agomelatine (25 mg/d), one received sertraline (50 mg/d) and escitalopram (10 mg/d), one received sertraline (50 mg/d) and venlafaxine (150 mg/d), one received sertraline (150 mg/d) and mirtazapine (15 mg/d), one received agomelatine (25 mg/d) and escitalopram (10 mg/d), one received fluoxetine (20 mg/d) and trazodone (25 mg/d), one received escitalopram (15 mg/d) and tandospirone (30 mg/d), and the remaining three received psychological therapy alone. The average fluoxetine equivalent dose was 39.80 mg, calculated as described [24]. Additionally, participants were permitted to receive additional medication such as hypnotics and sedatives.

The primary outcome was remission of depressive symptoms, defined as BDI ≤ 10, immediately after 6-week treatment.

### Genome-wide methylation

From the participants of the mentioned main study, four female MDD patients and five controls (three boys and two girls) were randomly selected for the genome-wide methylation analysis. Whole-blood samples were collected for PCR in 3-ml EDTA tubes. Genomic DNA was extracted from blood samples (200 μl) using the QIAamp DNA Kit (Qiagen, Hilden, Germany). The bisulfite-modified DNA was purified before performing the PCR. DNA libraries were constructed through PCR amplification and DNA fragmentation, after which they were hybridized on 850 K Illumina Infinium MethylationEPIC BeadChip arrays and scanned using the iScan System (Illumina, San Diego, USA).

We used the “limma” package in R to create a linear model to identify differences in methylation, whose significance we assessed after adjusting for multiple testing using the Benjamini–Hochberg method. Differentially methylated sites (probes) were filtered based on an p < 0.01. Differentially expressed DNA methylations were functionally enriched using Kyoto Encyclopedia of Genes and Genome database (KEGG) [25]. Fisher test is used to test the significance of enrichment (*p* < 0.01).

### RPS6KA5 methylation analysis

RPS6KA5 methylation was analyzed using DNA samples from all MDD patients. The methylation reaction primers in the RPS6KA5 were designed using the PyroMark Assay design Software 2.0 (Qiagen, Hilden, Germany), and they had the following sequences: 5'-TTTGGAGATGATGGTTTTAGAAATTTAAAG-3', 5'-TCCAAAATAAACAACCTAAACCTACACCA-3', and 5'-ATTTAAAGTTTTAGGTAAAAGGA-3'.

PCR amplification of bisulfite- modified DNA was performed as follows. A 50-μl mixture was prepared for each reaction, which included 10 μl 5X GC buffer (KAPA), 10 mM dNTP, 50 pM upstream primer, 50 pM downstream primer, 2 μl template DNA, and 1 U/μl Taq polymerase. The cycling program began with 95 °C for 3 min, followed by 40 cycles at 94 °C for 30 s, 56 °C for 30 s, and 72 °C for 1 min, then finally 72 °C for 7 min. Samples were measured with the PyroMark Gold Q96 Reagent Kit (Qiagen) on a PSQ 96 ID Pyrosequencer (Qiagen), and analyzed using Pyro-Q-CpG 1.0.9 software (Qiagen). The methylation level at each CpG site was calculated as the percentage of the methylated alleles over the sum of methylated and unmethylated alleles. The mean methylation level was calculated using methylation levels of all measured CpG sites within each targeted region.

### Statistical analysis

All statistical analyses were performed using SPSS 26.0 (IBM, Armonk, NY, USA). Differences associated with a two-tailed p < 0.05 were considered statistically significant. To explore the associations between demographic and clinical variables, we conducted independent sample t-tests, chi-squared tests, and bivariate correlation analyses. Logistic and linear regression analyses were carried out to determine whether chronic childhood stress can predict the occurrence and severity of depression, and whether RPS6KA5 methylation can predict subsequent improvement in depressive symptoms after treatment.

## Results

### Genome-wide methylation analysis

**Fig. 1:**
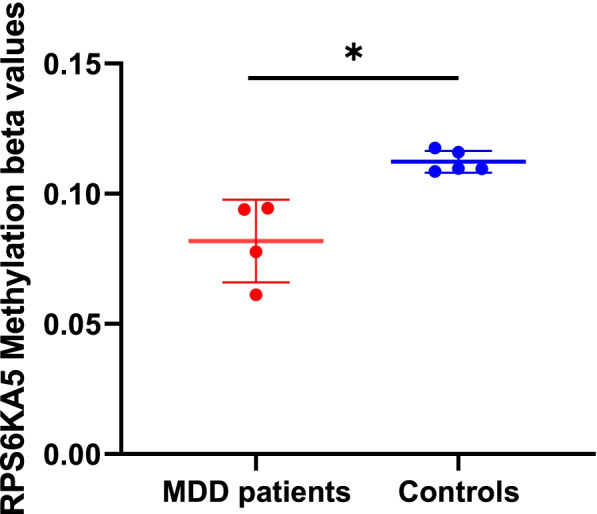
RPS6KA5 methylation beta values of MDD patients and controls. Values are expressed as means ± standard errors

In four MDD patients and five controls, we found 3034 DNA methylations that were differentially expressed. Compared with healthy controls, 1028 genes were demethylated and 2006 genes were methylated in patients **(**Supplementary Figs. 1 and 2**)**. RPS6KA5 was one of those differentially methylated genes (log(fold change) = 0.46, *p* = 2.74E-03), which also showed the highest expression in the human brain as part of the neurotrophic factor cascades (Supplementary Figs. 3, 4 and 5). The mean beta value of MDD patients was 0.08 and the mean beta value HC was 0.11 (Fig. 1). Therefore, subsequent experiments focused on RPS6KA5.

### Demographic and clinical characteristics

Among a total of 87 MDD patients who underwent a 6-week antidepressant trial, only three patients showed no signs of remission based on their BDI scores. There were no significant differences between the MDD patients and controls in age, sex, or level of education. At baseline, MDD patients showed significantly higher CCSQ scores than controls (Table 1).

**Table 1:** Demographic and clinical characteristics of MDD patients and healthy controls at baseline

Characteristic	MDD patients (*n* = 87)	Controls (*n* = 53)	*F* or *χ*^*2*^	*p*
**Demographic characteristics**				
Age (years)	14.47 ± 1.47	14.21 ± 1.36	0.181	0.292
Sex (Male/Female)	15/72	14/39	1.688	0.194
Level of education (Junior/Senior)	50/37	39/14	3.693	0.055
**Clinical characteristics**				
CCSQ total score	137.78 ± 39.56	83.02 ± 22.97	18.984	< 0.001
Peer bullying	28.60 ± 12.25	19.51 ± 6.41	30.112	< 0.001
Childhood abuse and neglect	64.07 ± 21.99	40.26 ± 14.42	11.182	< 0.001
Adverse childhood experiences	45.11 ± 13.23	23.25 ± 6.95	23.194	< 0.001
BDI score	34.24 ± 8.78	7.13 ± 6.61	1.425	< 0.001

Values are expressed as n or as means ± standard errors, unless otherwise indicated. Raw data are in Supplementary Table 1

### Effect of chronic childhood stress on risk and severity of depression

**Fig. 2:**
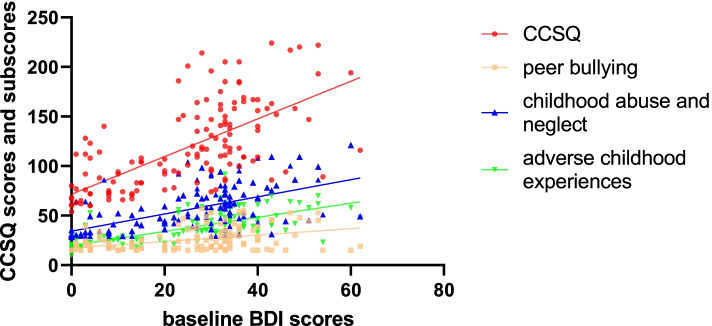
The relationship between BDI and CCSQ scores

There was a significant relationship between BDI and CCSQ scores in the samples (*r* = 0.677, *p* < 0.001), but the strength of the correlation varied among the three types of stressors (Fig. 2). Baseline BDI score strongly correlated with CCSQ subscores for adverse childhood experiences (*r* = 0.709, *p* < 0.001), while it moderately correlated with the CCSQ subscore for childhood abuse and neglect (*r* = 0.591, *p* < 0.001) and peer bullying (*r* = 0.442, *p* < 0.001).

Binary logistic regression showed that the severity of chronic childhood stress was significantly associated with the risk of depression (OR 0.943, 95% confidence interval (CI) 0.924–0.962, *p* < 0.001). The *p* value based on the Hosmer–Lemeshow test was 0.151.

Among the MDD patients, when we performed multiple linear regression using baseline BDI scores as the dependent variable (R2 = 0.236, *F* = 6.319, *p* < 0.001; Fig. 2), we found a significant association with CCSQ score (standardized β = 0.45, *p* < 0.001) and age (β =—0.361, *p* = 0.027), but no association with sex (β = 0.13, *p* = 0.185) or level of education (β = 0.058, *p* = 0.71).

### RPS6KA5 methylation was not associated with chronic childhood stress and severity of depression in MDD patients

**Fig. 3:**
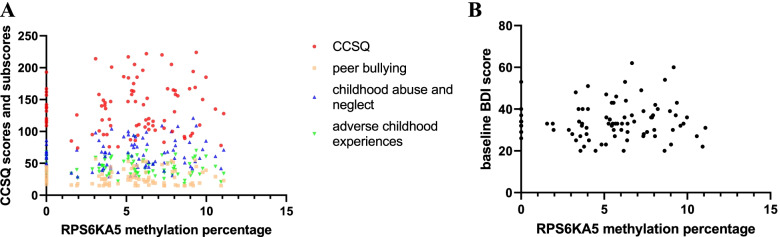
**A** The relationship between RPS6KA5 methylation percentage and CCSQ scores. **B** The relationship between RPS6KA5 methylation percentage and baseline BDI scores

The analyzed sequence contains 3 CpGs within the RPS6KA5. The average methylation percentage among 3 CpGs of all 87 MDD patients was 5.30 ± 3.01. We observed that RPS6KA5 methylation was not associated with chronic childhood stress or severity of depression (*p* > 0.05) (Fig. 3 A and B).

### RPS6KA5 methylation predicts remission of MDD patients after six weeks of treatment

**Fig. 4:**
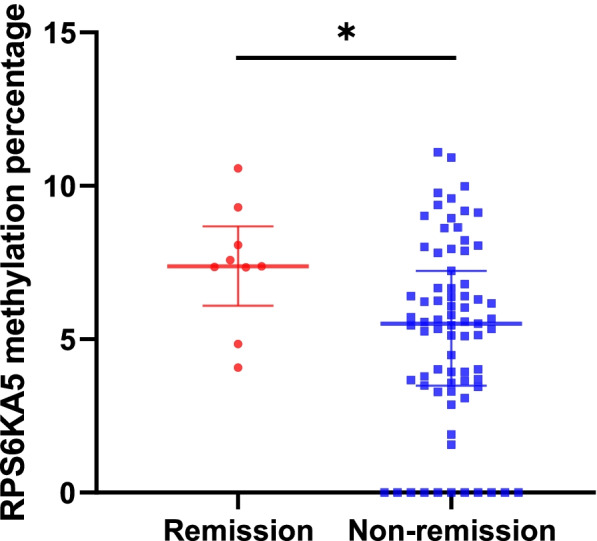
RPS6KA5 methylation percentages of MDD patients stratified by whether they experienced remission or not. Values are expressed as means ± standard errors

Among 84 MDD patients who completed follow-up, BDI score fell from 34.44 ± 8.87 at baseline to 27.80 ± 12.78 after six weeks of treatment (t = 5.616, *p* < 0.001). Nine achieved remission, as defined by BDI ≤ 10 (Table 2). There were no significant differences in any clinicodemographic characteristics, except RPS6KA5 methylation, between the patients who achieved remission and those who did not (Table 2 and Fig. 4).

**Table 2:** Demographic and clinical characteristics of MDD patients stratified by whether they experienced remission or not

Characteristic	Remission (*n* = 9)	Non-remission (*n* = 75)	F or *χ*^*2*^	*p*
**Demographic characteristics**				
Age (years)	15.00 ± 1.32	14.45 ± 1.47	0.249	0.291
Sex (Male/Female)	3/6	11/64	2.016	0.168
Level of education (Junior/Senior)	5/4	43/32	0.01	1
**Clinical characteristics**				
CCSQ total score	146.78 ± 39.28	137.41 ± 39.81	0.004	0.506
Peer bullying	30.56 ± 15.44	28.34 ± 11.99	1.798	0.613
Childhood abuse and neglect	68.00 ± 20.72	63.98 ± 22.23	0.451	0.607
Adverse childhood experience	48.22 ± 13.07	45.10 ± 13.35	0.000	0.508
Baseline BDI score	30.78 ± 6.76	34.88 ± 9.03	0.213	0.191
Antidepressant dose^a^ (mg)	36.94 ± 11.94	40.15 ± 16.61	1.354	0.577
RPS6KA5 methylation percentage	7.39 ± 1.99	5.19 ± 2.99	2.825	0.035

^a^ The antidepressant dose was calculated in fluoxetine equivalents

Values are expressed as n or as means ± standard errors, unless indicated otherwise. Raw data are in Supplementary Table 2

Binary logistic regression with antidepressant dose as covariates revealed that the RPS6KA5 methylation percentage at baseline significantly predicted remission at 6-week follow-up (OR 1.354, 95% CI 1.010–1.817, *p* = 0.043). The *p* value for the Hosmer–Lemeshow test was 0.454.

## Discussion

In the present study, we investigated the effect of RPS6KA5 methylation and chronic childhood stress on depressive symptoms in adolescents, and we examined whether RPS6KA5 methylation can predict remission after six weeks of treatment. RPS6KA5 was selected as a target gene based on genome-wide DNA methylation analysis. Our findings show that the severity of chronic childhood stress is associated with increased risk of depression in adolescents, and baseline RPS6KA5 methylation may be useful for predicting remission after 6-week treatment. We did not observe any interaction between RPS6KA5 methylation and chronic childhood stress in influencing the severity of depression.

Several studies have highlighted that childhood adversity is a risk factor for depression in adolescents [26–29], and our results are consistent with those studies. Of the three dimensions used to measure chronic stress, we found that adverse childhood experiences (including family-related, school-related, and individual experiences) were significantly associated with self-reported depression symptoms, followed by childhood abuse and neglect. Interventions to reduce stress may help decrease the risk of MDD. Additionally, positive parenting practices and peer social support can provide potentially valuable resilience strategies [30, 31].

In recent years, DNA methylation has received wide attention as a crucial epigenetic regulatory mechanism. At the same time, with the widespread use of whole genome bisulfite sequencing technology and bioinformatics analysis, a large number of differentially methylated genes have been identified. Therefore, in our study, we used a high-throughput methylation array and selected four adolescents with MDD and five healthy controls. As a result, we found 3034 DNA methylations that were differentially expressed in MDD patients and HC and selected RPS5KA as gene of interest. KEGG pathway annotation showed that RPS5KA participated in neurotrophin signaling pathways, which was considered to be sensitive to inflammatory response and stress activity. Previous studies also regarded the interaction of childhood adversity with genotypes as a significant contributor to methylation variability [6, 32]. However, correlation analysis did not reveal significant correlations between chronic childhood stress and RPS6KA5 methylation percentage. Further research will be needed to explicitly elucidate the possible interactions with environmental factors.

An important observation was that patients with lower methylation percentage had an even worse outcome. As far as we know, this is the first study to identify an association between RPS5KA methylation and response to treatment for adolescent MDD. Accompanied by the monoamine theory, BDNF was considered to be required for antidepressant response [33]. RPS5KA is a downstream enzyme of BDNF that regulates gene expression and therefore may participate in the pathogenesis of depression [34]. In a recent review, the author suggested that RPS5KA might regulate key plasticity-related genes through epigenetic mechanisms to potentially avoid depression, which also supported our results [35]. As part of this neurotrophic factor cascades, MAPK signaling pathway and TNF signaling pathway might play a crucial role in immune and inflammatory response [36, 37]. This agrees with the conclusions from previous studies that strong inflammation in MDD patients might be associated with drug resistance [38].

An interesting finding from our study is that RPS5KA methylation did not appear to affect the severity of depressive symptoms, although it had a significant effect on response to treatment. Previous studies have linked inflammation to specific symptoms such as atypical depressive symptoms [39], but this has been less studied in adolescents with depression [40]. The heterogeneity of depression could obscure possible associations with RPS5KA methylation, so further research into MDD may help clarify the situation.

There exist a couple of limitations in the research. First, the sample size of participants used for the genome-wide DNA methylation was relatively small and sex was not considered. Additional study with a larger sample size is warranted. Second, we only performed targeted sequencing in patient so we cannot conclude whether altered RPS5KA methylation was specific to antidepressant response. However, the purpose of this study was to explore predictor and prognostic biomarker, and we considered the conclusion could be drawn from the available data. Third, peripheral blood samples may not represent methylation processes in the brain, and heterogeneity of white blood cell types has potential confounding effects. Fourth, self-report rating scales eliminated observer bias but may introduce reporting bias. Additionally, there was no standardized treatment in all patients, which remained a possible confounder but may also make the results more generalizable in clinical practice.

## Conclusion

The major finding to emerge from this study is that RPS6KA5 methylation has the potential to be used as a predictor of early response to treatment in adolescent MDD patients. Additionally, chronic childhood stress is again confirmed closely related to the occurrence and development of depression symptoms. Prior to this investigation, there is no report on the involvement of RPS6KA5 gene in MDD. Further research is required to replicate the present findings and develop a deeper understanding of the relationship between epigenetics and response to treatment of depression.

## Supplementary Information

**Additional file 1: Supplementary Figure SFig. 1. **DNA methylations differentially expressed between MDD patients and HC,red indicate genes that were differentially expressed. Raw data can be directed to the corresponding author.**Supplementary Figure SFig. 2.**Hierarchical clustering of DNA methylations between MDD patients and HC. The results show different patterns between the two groups and homogeneity within each group. Red and blue indicate up- or down-regulation (methylated or demethylated), respectively. Raw data can be directed to the corresponding author.**Supplementary Figure SFig. 3. **KEGG pathway for Neurotrophin signaling pathway. **Supplementary Figure SFig. 4. **KEGG pathway for MAPK signaling pathway. **Supplementary Figure SFig. 5. **KEGG pathway for TNF signaling pathway.

**Additional file 2: Supplementary Table STab. 1. **Raw data of Table 1. Demographic and clinical characteristics of MDD patients and healthy controls at baseline.

**Additional file 3: Supplementary Table STab. 2. **Raw data of Table 2. Demographic and clinical characteristics of MDD patients stratified by whether they experienced remission or not.

## Data Availability

The original contributions presented in the study are included in the article/Supplementary Material. Further inquiries can be directed to the corresponding author (Li Yin, yli009@163.com). A part of data are not publicly available due to privacy or ethical restrictions.

## Abbreviations

BDI: Beck Depression Inventory-II
CCSQ: Childhood Chronic Stress Questionnaire
HC: Healthy controls
KEGG: Kyoto Encyclopedia of Genes and Genome database
MDD: Major Depressive Disorder
SPSS: Statistical Product and Service Solutions
