# Supplementary material for: RPS6KA5 methylation predict response to 6-week treatment for adolescent MDD patients
Source: BMC Psychiatry. 2022 Aug 19;22:561. doi: 10.1186/s12888-022-04196-4 (PMC9392312; doi:10.1186/s12888-022-04196-4)
Supplement: Supplementary file 1 — Additional file 1: Supplementary Figure SFig. 1. DNA methylations differentially expressed between MDD patients and HC,red indicate genes that were differentially expressed. Raw data can be directed to the corresponding author.Supplementary Figure SFig. 2.Hierarchical clustering of DNA methylations between MDD patients and HC. The results show different patterns between the two groups and homogeneity within each group. Red and blue indicate up- or down-regulation (methylated or demethylated), respectively. Raw data can be directed to the corresponding author.Supplementary Figure SFig. 3. KEGG pathway for Neurotrophin signaling pathway. Supplementary Figure SFig. 4. KEGG pathway for MAPK signaling pathway. Supplementary Figure SFig. 5. KEGG pathway for TNF signaling pathway. [file 12888_2022_4196_MOESM1_ESM.docx]

Supplementary Material for

**RPS6KA5 methylation predict response to 6-week treatment for adolescent MDD patients**

**This supplement contains:**

- Supplementary figure and table legends (this file)
- Supplementary Figure SFig. 1
- Supplementary Figure SFig. 2
- Supplementary Figure SFig. 3
- Supplementary Figure SFig. 4
- Supplementary Figure SFig. 5
- Supplementary Table STab. 1
- Supplementary Table STab. 2

**Supplementary Figure legend**

**Supplementary Figure SFig.1. DNA methylations differentially expressed between MDD patients and HC, red indicate genes that were differentially expressed.** Raw data can be directed to the corresponding author.


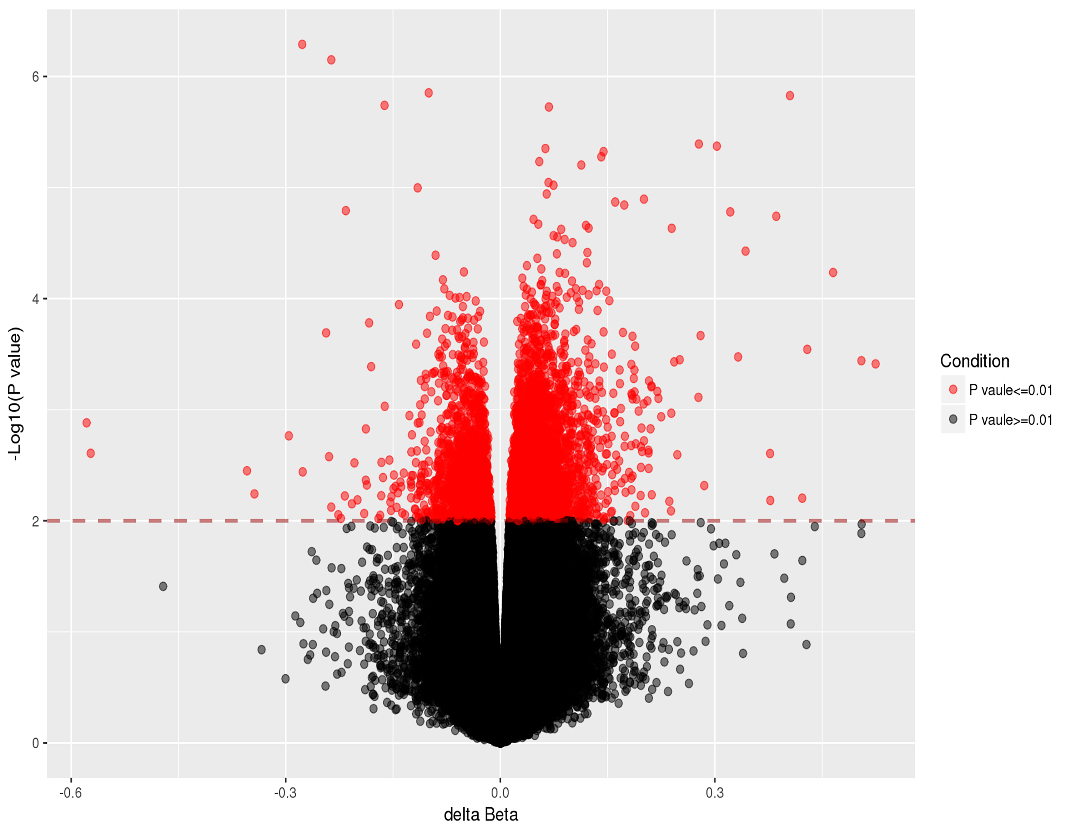


**Supplementary Figure SFig.2.** **Hierarchical clustering of DNA methylations between MDD patients and HC.** The results show different patterns between the two groups and homogeneity within each group. Red and blue indicate up- or down-regulation (methylated or demethylated), respectively. Raw data can be directed to the corresponding author.


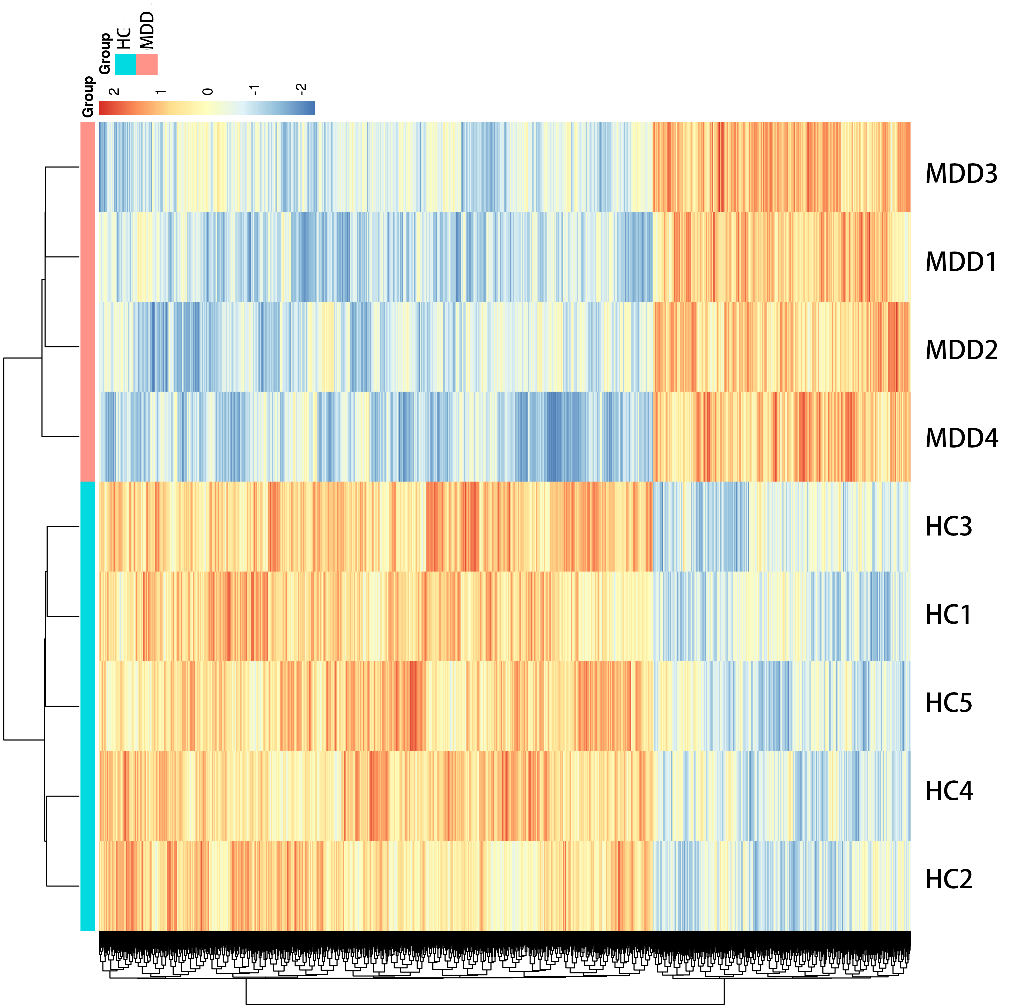


**Supplementary Figure SFig.3. KEGG pathway for Neurotrophin signaling pathway**

**
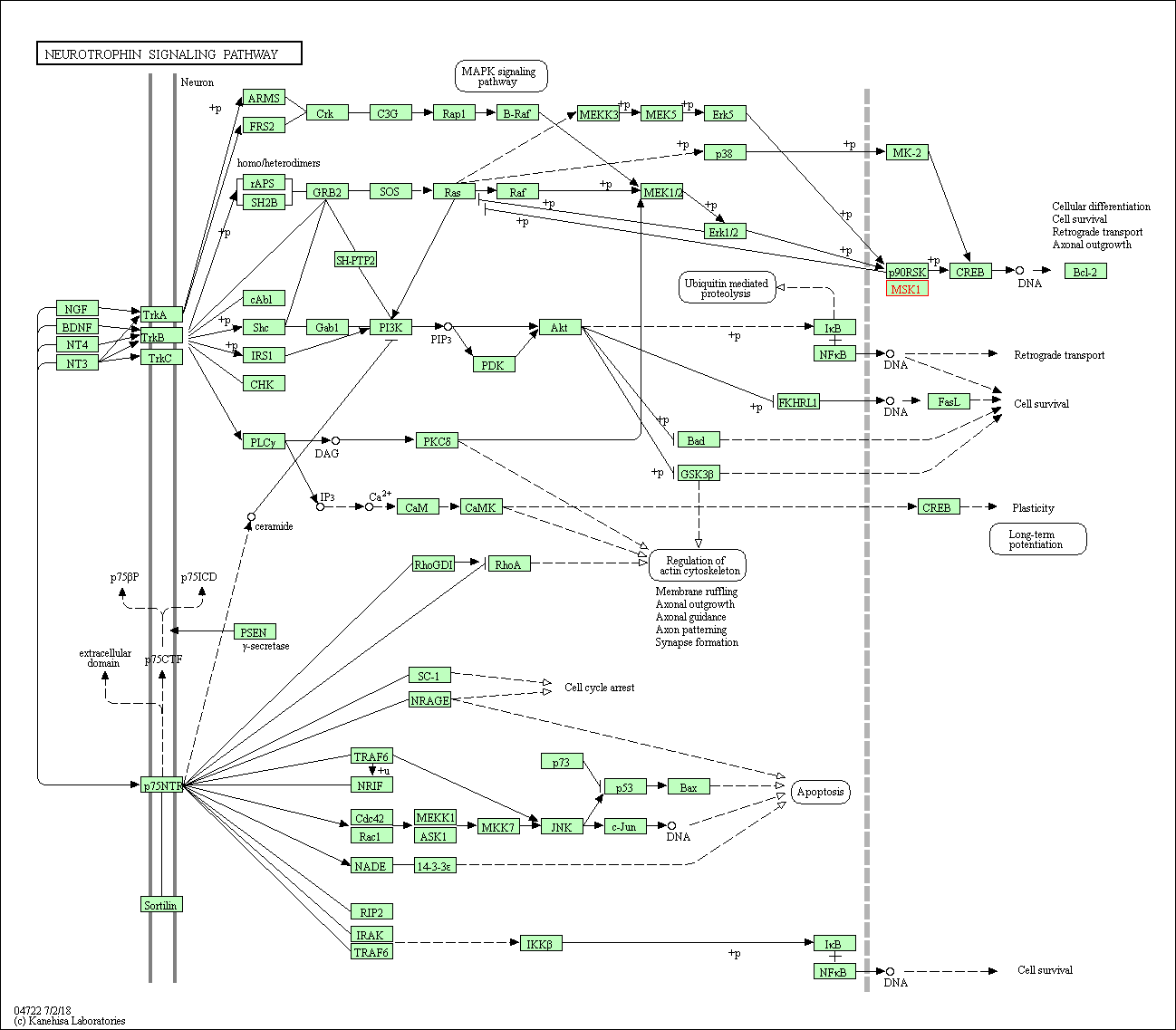
**

**Supplementary Figure SFig.4. KEGG pathway for MAPK signaling pathway**

**
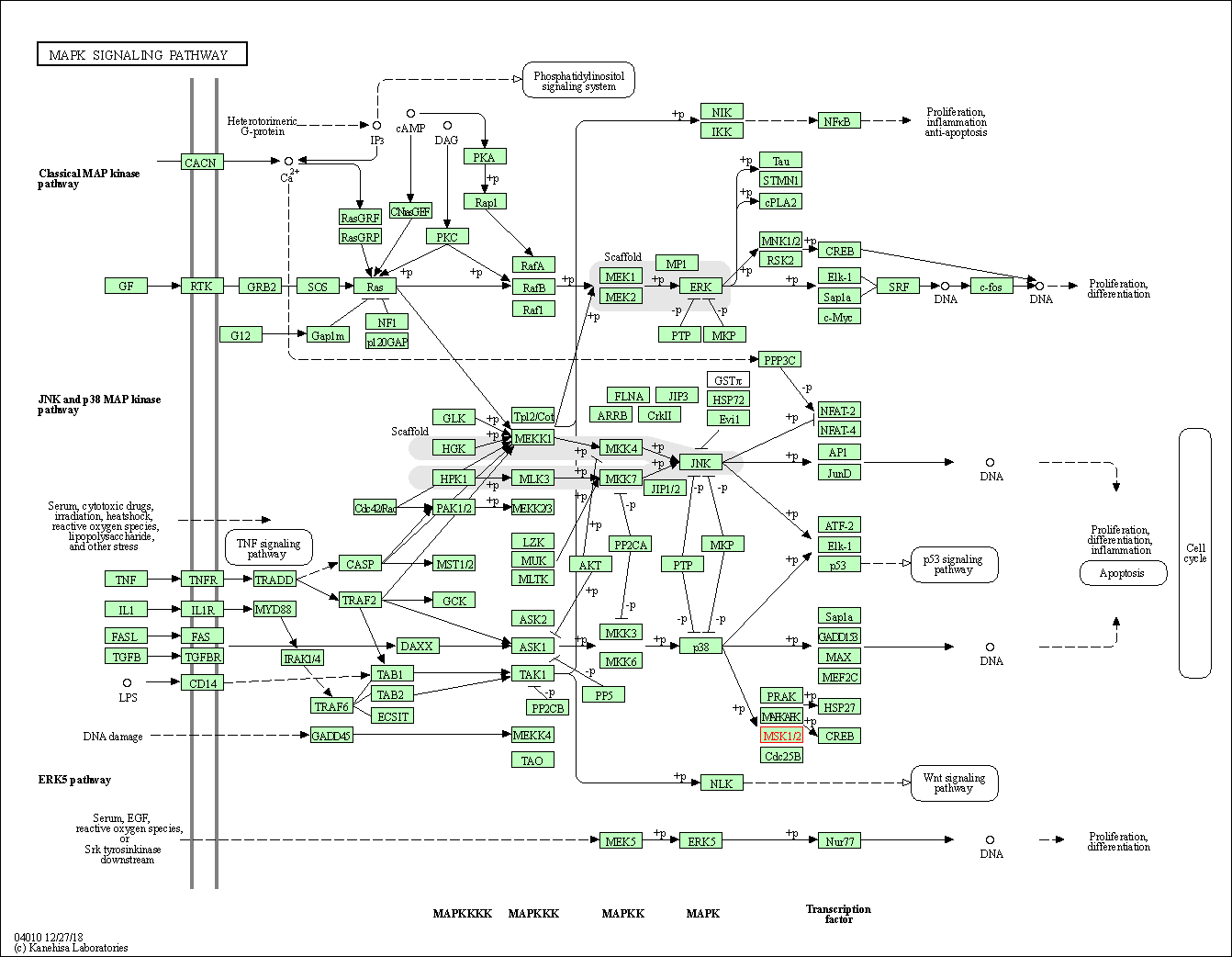
**

**Supplementary Figure SFig.5. KEGG pathway for TNF signaling pathway**


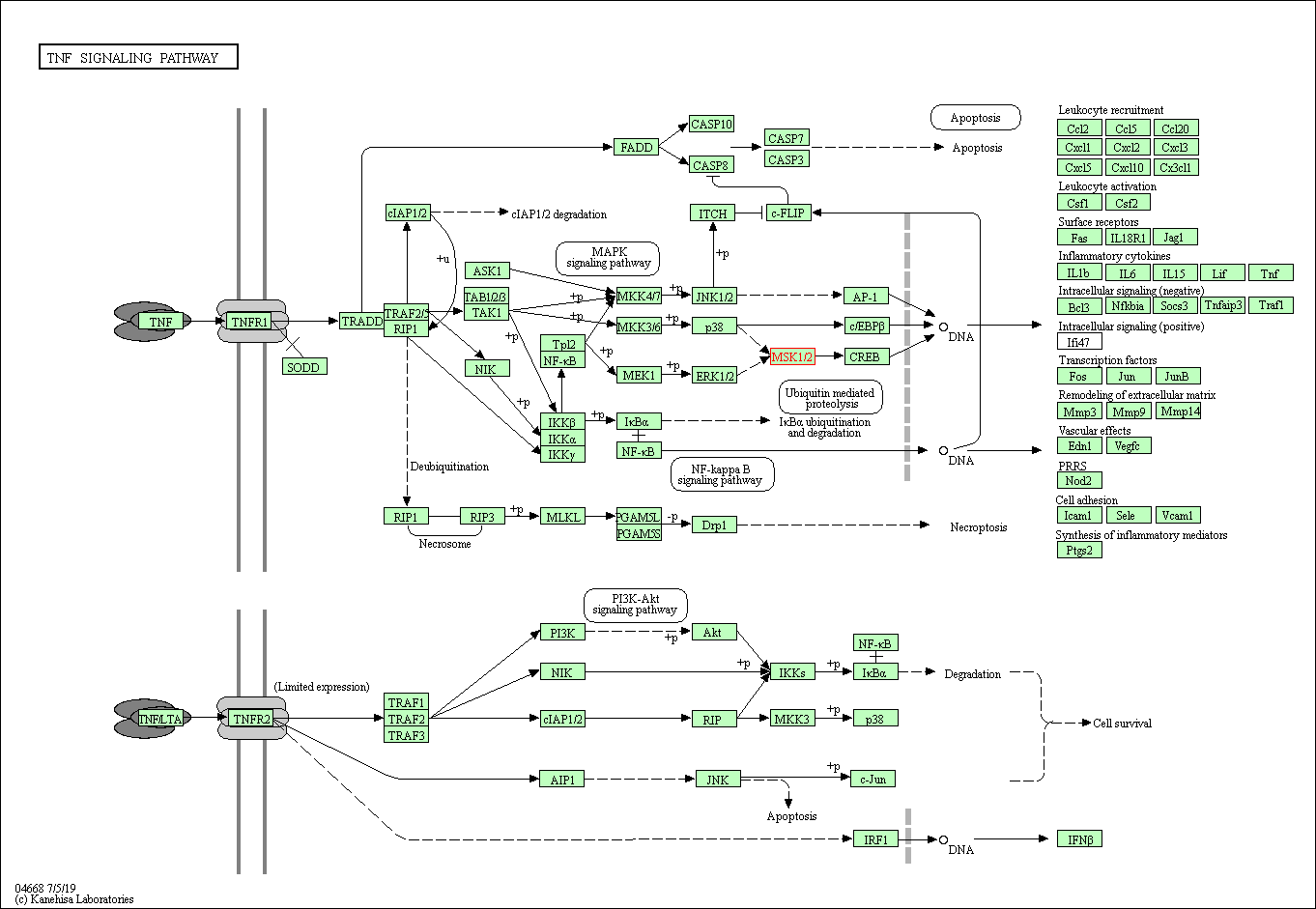


**Supplementary Table legends**

**Supplementary Table STab. 1. Raw data of Table 1. Demographic and clinical characteristics of MDD patients and healthy controls at baseline.**

| group(1=MDD patients,0=Controls) | sex | age | level of education | CCSQ | peer bullying | childhood abuse and neglect | adverse childhood experiences | BDI w0 | BDI w6 | RPS6KA5 methylation percentage |
| --- | --- | --- | --- | --- | --- | --- | --- | --- | --- | --- |
| 1 | 2 | 12 | 1 | 116 | 19 | 49 | 48 | 62 | 58 | 6.67 |
| 1 | 1 | 12 | 1 | 109 | 21 | 49 | 39 | 37 | 21 | 0 |
| 1 | 2 | 12 | 1 | 193 | 45 | 86 | 62 | 53 | 53 | 0 |
| 1 | 2 | 12 | 1 | 118 | 36 | 47 | 35 | 36 | 30 | 6.22 |
| 1 | 2 | 12 | 1 | 95 | 17 | 48 | 30 | 22 | 24 | 3.79 |
| 1 | 2 | 12 | 1 | 85 | 16 | 35 | 34 | 33 | 41 | 1.56 |
| 1 | 2 | 12 | 1 | 164 | 42 | 62 | 60 | 35 | 22 | 5.34 |
| 1 | 1 | 12 | 1 | 137 | 43 | 58 | 36 | 29 | withdraw | 0 |
| 1 | 1 | 13 | 1 | 131 | 28 | 58 | 45 | 30 | 35 | 8.01 |
| 1 | 2 | 13 | 1 | 156 | 15 | 108 | 33 | 40 | 28 | 8.22 |
| 1 | 2 | 13 | 1 | 165 | 52 | 71 | 42 | 37 | 35 | 7.94 |
| 1 | 2 | 13 | 1 | 87 | 24 | 30 | 33 | 33 | 36 | 5.26 |
| 1 | 2 | 13 | 1 | 102 | 19 | 53 | 30 | 34 | 31 | 6.8 |
| 1 | 2 | 13 | 1 | 194 | 15 | 121 | 58 | 60 | 53 | 9.18 |
| 1 | 2 | 13 | 1 | 157 | 42 | 47 | 68 | 32 | 44 | 0 |
| 1 | 2 | 13 | 1 | 83 | 19 | 38 | 26 | 27 | 5 | 7.35 |
| 1 | 2 | 13 | 1 | 95 | 18 | 36 | 41 | 30 | 44 | 2.87 |
| 1 | 2 | 13 | 1 | 78 | 15 | 42 | 21 | 22 | 13 | 10.92 |
| 1 | 2 | 13 | 1 | 100 | 37 | 31 | 32 | 36 | 28 | 7.88 |
| 1 | 2 | 13 | 1 | 103 | 16 | 52 | 35 | 29 | 24 | 6.4 |
| 1 | 2 | 13 | 1 | 118 | 33 | 39 | 46 | 34 | 23 | 3.49 |
| 1 | 2 | 13 | 1 | 109 | 29 | 43 | 37 | 40 | 32 | 4.02 |
| 1 | 2 | 13 | 1 | 117 | 25 | 48 | 44 | 29 | 12 | 0 |
| 1 | 2 | 13 | 1 | 76 | 18 | 36 | 22 | 20 | 39 | 4.48 |
| 1 | 2 | 13 | 1 | 74 | 18 | 29 | 27 | 30 | withdraw | 1.95 |
| 1 | 2 | 14 | 1 | 205 | 33 | 80 | 92 | 33 | 24 | 5.51 |
| 1 | 1 | 14 | 1 | 169 | 55 | 53 | 61 | 37 | 8 | 9.3 |
| 1 | 2 | 14 | 1 | 145 | 24 | 61 | 60 | 33 | 28 | 3.67 |
| 1 | 2 | 14 | 2 | 95 | 15 | 42 | 38 | 20 | 17 | 3.57 |
| 1 | 1 | 14 | 1 | 136 | 26 | 67 | 43 | 40 | 34 | 3.7 |
| 1 | 1 | 14 | 1 | 217 | 53 | 100 | 64 | 47 | 33 | 5.12 |
| 1 | 2 | 14 | 2 | 76 | 15 | 29 | 32 | 31 | 45 | 3.94 |
| 1 | 2 | 14 | 1 | 167 | 39 | 67 | 61 | 40 | 37 | 0 |
| 1 | 1 | 14 | 1 | 111 | 25 | 57 | 29 | 33 | 33 | 5.79 |
| 1 | 2 | 14 | 1 | 222 | 53 | 99 | 70 | 53 | 53 | 6.25 |
| 1 | 2 | 14 | 1 | 120 | 37 | 39 | 44 | 26 | 39 | 0 |
| 1 | 2 | 14 | 1 | 105 | 15 | 51 | 39 | 39 | 0 | 7.36 |
| 1 | 2 | 14 | 1 | 94 | 15 | 44 | 35 | 46 | 28 | 5.72 |
| 1 | 2 | 14 | 1 | 175 | 28 | 94 | 53 | 32 | 37 | 5.45 |
| 1 | 2 | 14 | 1 | 110 | 19 | 58 | 33 | 32 | 28 | 9.59 |
| 1 | 2 | 14 | 1 | 167 | 49 | 68 | 50 | 29 | 5 | 7.58 |
| 1 | 2 | 14 | 1 | 114 | 22 | 50 | 42 | 34 | 23 | 0 |
| 1 | 2 | 14 | 1 | 163 | 46 | 75 | 42 | 43 | 40 | 5.45 |
| 1 | 2 | 14 | 1 | 89 | 15 | 51 | 23 | 54 | 30 | 8.65 |
| 1 | 2 | 14 | 1 | 167 | 35 | 83 | 49 | 39 | 33 | 8.95 |
| 1 | 2 | 15 | 2 | 117 | 15 | 67 | 35 | 28 | 0 | 7.38 |
| 1 | 1 | 15 | 1 | 107 | 21 | 44 | 42 | 20 | 15 | 6.17 |
| 1 | 2 | 15 | 1 | 150 | 36 | 72 | 42 | 33 | 28 | 9.77 |
| 1 | 2 | 15 | 2 | 120 | 28 | 44 | 48 | 33 | 21 | 6.4 |
| 1 | 2 | 15 | 1 | 185 | 46 | 76 | 63 | 33 | 33 | 5.67 |
| 1 | 2 | 15 | 2 | 147 | 44 | 45 | 58 | 51 | 37 | 4.02 |
| 1 | 2 | 15 | 2 | 141 | 18 | 67 | 56 | 32 | 42 | 3.65 |
| 1 | 2 | 15 | 1 | 140 | 25 | 68 | 47 | 40 | 36 | 3.45 |
| 1 | 2 | 15 | 2 | 220 | 48 | 109 | 63 | 49 | 48 | 7.23 |
| 1 | 1 | 15 | 2 | 109 | 22 | 49 | 38 | 37 | 29 | 6.66 |
| 1 | 2 | 15 | 2 | 86 | 15 | 31 | 40 | 43 | 43 | 5.56 |
| 1 | 2 | 15 | 1 | 126 | 15 | 71 | 40 | 33 | 25 | 1.9 |
| 1 | 2 | 15 | 2 | 152 | 22 | 80 | 50 | 44 | 36 | 6.3 |
| 1 | 2 | 15 | 2 | 158 | 30 | 79 | 49 | 35 | 39 | 5.34 |
| 1 | 2 | 15 | 2 | 147 | 24 | 67 | 56 | 23 | 26 | 5.1 |
| 1 | 2 | 15 | 2 | 142 | 24 | 63 | 55 | 33 | 23 | 6.07 |
| 1 | 2 | 15 | 1 | 196 | 53 | 90 | 53 | 30 | 20 | 6.03 |
| 1 | 1 | 15 | 2 | 112 | 24 | 52 | 36 | 25 | 18 | 6.38 |
| 1 | 2 | 15 | 2 | 149 | 26 | 77 | 46 | 27 | withdraw | 3.56 |
| 1 | 2 | 16 | 2 | 158 | 16 | 78 | 64 | 42 | 10 | 8.07 |
| 1 | 2 | 16 | 2 | 224 | 45 | 109 | 70 | 43 | 49 | 9.37 |
| 1 | 2 | 16 | 2 | 205 | 54 | 100 | 51 | 36 | 22 | 7.82 |
| 1 | 2 | 16 | 2 | 158 | 30 | 86 | 42 | 48 | 19 | 3.29 |
| 1 | 2 | 16 | 2 | 93 | 17 | 45 | 31 | 23 | 19 | 9.02 |
| 1 | 2 | 16 | 2 | 119 | 19 | 66 | 34 | 28 | 34 | 3.94 |
| 1 | 2 | 16 | 2 | 109 | 27 | 48 | 34 | 27 | 23 | 5.64 |
| 1 | 2 | 16 | 1 | 135 | 29 | 61 | 45 | 27 | 2 | 10.57 |
| 1 | 2 | 16 | 2 | 92 | 15 | 59 | 18 | 20 | 13 | 8.63 |
| 1 | 2 | 16 | 2 | 147 | 19 | 92 | 36 | 30 | 35 | 5.57 |
| 1 | 2 | 16 | 2 | 162 | 32 | 80 | 50 | 32 | 29 | 0 |
| 1 | 2 | 16 | 2 | 128 | 23 | 71 | 34 | 31 | 20 | 11.09 |
| 1 | 1 | 16 | 2 | 186 | 41 | 92 | 53 | 23 | 3 | 4.84 |
| 1 | 1 | 16 | 2 | 138 | 29 | 64 | 45 | 34 | 22 | 0 |
| 1 | 2 | 17 | 2 | 184 | 29 | 95 | 60 | 36 | 35 | 5.13 |
| 1 | 1 | 17 | 2 | 201 | 36 | 104 | 61 | 25 | 5 | 4.08 |
| 1 | 1 | 17 | 2 | 97 | 19 | 42 | 36 | 30 | 22 | 9.13 |
| 1 | 1 | 17 | 2 | 164 | 38 | 86 | 40 | 27 | 16 | 8.05 |
| 1 | 2 | 17 | 2 | 132 | 17 | 69 | 46 | 34 | 30 | 0 |
| 1 | 2 | 17 | 2 | 185 | 39 | 85 | 61 | 36 | 23 | 9.98 |
| 1 | 2 | 17 | 2 | 142 | 25 | 60 | 57 | 34 | 32 | 0 |
| 1 | 2 | 17 | 2 | 101 | 15 | 49 | 37 | 25 | 21 | 3.3 |
| 1 | 2 | 17 | 2 | 214 | 57 | 98 | 59 | 28 | 26 | 3.08 |
| 0 | 2 | 12 | 2 | 62 | 15 | 29 | 18 | 2 |  |  |
| 0 | 2 | 12 | 2 | 61 | 15 | 28 | 18 | 0 |  |  |
| 0 | 2 | 12 | 1 | 80 | 15 | 35 | 30 | 0 |  |  |
| 0 | 2 | 12 | 1 | 123 | 21 | 49 | 53 | 4 |  |  |
| 0 | 2 | 12 | 1 | 104 | 23 | 50 | 31 | 13 |  |  |
| 0 | 2 | 13 | 1 | 108 | 17 | 62 | 29 | 4 |  |  |
| 0 | 2 | 13 | 1 | 102 | 29 | 47 | 26 | 19 |  |  |
| 0 | 2 | 13 | 1 | 75 | 20 | 32 | 23 | 8 |  |  |
| 0 | 2 | 13 | 1 | 65 | 15 | 31 | 19 | 2 |  |  |
| 0 | 2 | 13 | 1 | 77 | 22 | 30 | 25 | 1 |  |  |
| 0 | 2 | 13 | 1 | 54 | 15 | 29 | 10 | 0 |  |  |
| 0 | 2 | 13 | 1 | 112 | 19 | 64 | 29 | 1 |  |  |
| 0 | 2 | 13 | 1 | 72 | 18 | 34 | 20 | 13 |  |  |
| 0 | 2 | 13 | 2 | 70 | 17 | 29 | 24 | 8 |  |  |
| 0 | 2 | 13 | 2 | 74 | 16 | 29 | 29 | 11 |  |  |
| 0 | 2 | 13 | 1 | 83 | 15 | 42 | 26 | 14 |  |  |
| 0 | 2 | 13 | 1 | 94 | 15 | 52 | 27 | 10 |  |  |
| 0 | 2 | 13 | 1 | 73 | 22 | 34 | 17 | 8 |  |  |
| 0 | 2 | 13 | 1 | 71 | 19 | 29 | 23 | 13 |  |  |
| 0 | 1 | 13 | 2 | 60 | 15 | 29 | 16 | 0 |  |  |
| 0 | 2 | 13 | 2 | 60 | 15 | 29 | 16 | 0 |  |  |
| 0 | 2 | 14 | 1 | 128 | 24 | 64 | 40 | 3 |  |  |
| 0 | 1 | 14 | 1 | 60 | 15 | 29 | 16 | 4 |  |  |
| 0 | 2 | 14 | 1 | 108 | 25 | 57 | 26 | 15 |  |  |
| 0 | 2 | 14 | 2 | 72 | 16 | 33 | 23 | 4 |  |  |
| 0 | 1 | 14 | 1 | 83 | 17 | 40 | 26 | 15 |  |  |
| 0 | 1 | 14 | 1 | 73 | 22 | 34 | 17 | 8 |  |  |
| 0 | 1 | 15 | 1 | 60 | 15 | 29 | 16 | 0 |  |  |
| 0 | 1 | 15 | 1 | 92 | 15 | 52 | 25 | 10 |  |  |
| 0 | 2 | 15 | 1 | 67 | 19 | 29 | 19 | 13 |  |  |
| 0 | 1 | 15 | 1 | 88 | 27 | 34 | 27 | 6 |  |  |
| 0 | 2 | 15 | 1 | 63 | 16 | 30 | 17 | 1 |  |  |
| 0 | 2 | 15 | 1 | 104 | 25 | 57 | 22 | 15 |  |  |
| 0 | 2 | 15 | 1 | 104 | 25 | 57 | 22 | 15 |  |  |
| 0 | 2 | 15 | 1 | 83 | 17 | 40 | 26 | 15 |  |  |
| 0 | 2 | 15 | 1 | 68 | 16 | 31 | 21 | 0 |  |  |
| 0 | 2 | 15 | 1 | 60 | 15 | 29 | 16 | 0 |  |  |
| 0 | 1 | 15 | 1 | 92 | 39 | 29 | 24 | 3 |  |  |
| 0 | 1 | 15 | 1 | 76 | 20 | 33 | 23 | 3 |  |  |
| 0 | 2 | 15 | 1 | 84 | 18 | 41 | 25 | 9 |  |  |
| 0 | 2 | 15 | 1 | 67 | 16 | 30 | 21 | 0 |  |  |
| 0 | 2 | 15 | 1 | 140 | 25 | 86 | 29 | 7 |  |  |
| 0 | 2 | 15 | 2 | 114 | 33 | 51 | 30 | 6 |  |  |
| 0 | 2 | 15 | 1 | 75 | 15 | 42 | 18 | 14 |  |  |
| 0 | 2 | 16 | 1 | 112 | 16 | 72 | 24 | 27 |  |  |
| 0 | 2 | 16 | 2 | 60 | 15 | 29 | 16 | 0 |  |  |
| 0 | 1 | 16 | 2 | 60 | 15 | 29 | 16 | 0 |  |  |
| 0 | 2 | 16 | 1 | 112 | 24 | 64 | 24 | 3 |  |  |
| 0 | 1 | 16 | 1 | 60 | 15 | 29 | 16 | 4 |  |  |
| 0 | 1 | 16 | 2 | 72 | 16 | 33 | 23 | 4 |  |  |
| 0 | 2 | 16 | 2 | 66 | 16 | 29 | 21 | 11 |  |  |
| 0 | 1 | 17 | 2 | 151 | 47 | 70 | 34 | 24 |  |  |
| 0 | 1 | 17 | 2 | 66 | 17 | 29 | 20 | 8 |  |  |

**Supplementary Table STab. 2. Raw data of Table 2. Demographic and clinical characteristics of MDD patients stratified by whether they experienced remission or not.**

| group(1=Remission,2=Non-remission) | sex | age | level of education | CCSQ | peer bullying | childhood abuse and neglect | adverse childhood experiences | BDI w0 | BDI w6 | RPS6KA5 methylation percentage |
| --- | --- | --- | --- | --- | --- | --- | --- | --- | --- | --- |
| 0 | 2 | 12 | 1 | 116 | 19 | 49 | 48 | 62 | 58 | 6.67 |
| 0 | 1 | 12 | 1 | 109 | 21 | 49 | 39 | 37 | 21 | 0 |
| 0 | 2 | 12 | 1 | 193 | 45 | 86 | 62 | 53 | 53 | 0 |
| 0 | 2 | 12 | 1 | 118 | 36 | 47 | 35 | 36 | 30 | 6.22 |
| 0 | 2 | 12 | 1 | 95 | 17 | 48 | 30 | 22 | 24 | 3.79 |
| 0 | 2 | 12 | 1 | 85 | 16 | 35 | 34 | 33 | 41 | 1.56 |
| 0 | 2 | 12 | 1 | 164 | 42 | 62 | 60 | 35 | 22 | 5.34 |
| withdraw | 1 | 12 | 1 | 137 | 43 | 58 | 36 | 29 |  | 0 |
| 0 | 1 | 13 | 1 | 131 | 28 | 58 | 45 | 30 | 35 | 8.01 |
| 0 | 2 | 13 | 1 | 156 | 15 | 108 | 33 | 40 | 28 | 8.22 |
| 0 | 2 | 13 | 1 | 165 | 52 | 71 | 42 | 37 | 35 | 7.94 |
| 0 | 2 | 13 | 1 | 87 | 24 | 30 | 33 | 33 | 36 | 5.26 |
| 0 | 2 | 13 | 1 | 102 | 19 | 53 | 30 | 34 | 31 | 6.8 |
| 0 | 2 | 13 | 1 | 194 | 15 | 121 | 58 | 60 | 53 | 9.18 |
| 0 | 2 | 13 | 1 | 157 | 42 | 47 | 68 | 32 | 44 | 0 |
| 1 | 2 | 13 | 1 | 83 | 19 | 38 | 26 | 27 | 5 | 7.35 |
| 0 | 2 | 13 | 1 | 95 | 18 | 36 | 41 | 30 | 44 | 2.87 |
| 0 | 2 | 13 | 1 | 78 | 15 | 42 | 21 | 22 | 13 | 10.92 |
| 0 | 2 | 13 | 1 | 100 | 37 | 31 | 32 | 36 | 28 | 7.88 |
| 0 | 2 | 13 | 1 | 103 | 16 | 52 | 35 | 29 | 24 | 6.4 |
| 0 | 2 | 13 | 1 | 118 | 33 | 39 | 46 | 34 | 23 | 3.49 |
| 0 | 2 | 13 | 1 | 109 | 29 | 43 | 37 | 40 | 32 | 4.02 |
| 0 | 2 | 13 | 1 | 117 | 25 | 48 | 44 | 29 | 12 | 0 |
| 0 | 2 | 13 | 1 | 76 | 18 | 36 | 22 | 20 | 39 | 4.48 |
| withdraw | 2 | 13 | 1 | 74 | 18 | 29 | 27 | 30 |  | 1.95 |
| 0 | 2 | 14 | 1 | 205 | 33 | 80 | 92 | 33 | 24 | 5.51 |
| 1 | 1 | 14 | 1 | 169 | 55 | 53 | 61 | 37 | 8 | 9.3 |
| 0 | 2 | 14 | 1 | 145 | 24 | 61 | 60 | 33 | 28 | 3.67 |
| 0 | 2 | 14 | 2 | 95 | 15 | 42 | 38 | 20 | 17 | 3.57 |
| 0 | 1 | 14 | 1 | 136 | 26 | 67 | 43 | 40 | 34 | 3.7 |
| 0 | 1 | 14 | 1 | 217 | 53 | 100 | 64 | 47 | 33 | 5.12 |
| 0 | 2 | 14 | 2 | 76 | 15 | 29 | 32 | 31 | 45 | 3.94 |
| 0 | 2 | 14 | 1 | 167 | 39 | 67 | 61 | 40 | 37 | 0 |
| 0 | 1 | 14 | 1 | 111 | 25 | 57 | 29 | 33 | 33 | 5.79 |
| 0 | 2 | 14 | 1 | 222 | 53 | 99 | 70 | 53 | 53 | 6.25 |
| 0 | 2 | 14 | 1 | 120 | 37 | 39 | 44 | 26 | 39 | 0 |
| 1 | 2 | 14 | 1 | 105 | 15 | 51 | 39 | 39 | 0 | 7.36 |
| 0 | 2 | 14 | 1 | 94 | 15 | 44 | 35 | 46 | 28 | 5.72 |
| 0 | 2 | 14 | 1 | 175 | 28 | 94 | 53 | 32 | 37 | 5.45 |
| 0 | 2 | 14 | 1 | 110 | 19 | 58 | 33 | 32 | 28 | 9.59 |
| 1 | 2 | 14 | 1 | 167 | 49 | 68 | 50 | 29 | 5 | 7.58 |
| 0 | 2 | 14 | 1 | 114 | 22 | 50 | 42 | 34 | 23 | 0 |
| 0 | 2 | 14 | 1 | 163 | 46 | 75 | 42 | 43 | 40 | 5.45 |
| 0 | 2 | 14 | 1 | 89 | 15 | 51 | 23 | 54 | 30 | 8.65 |
| 0 | 2 | 14 | 1 | 167 | 35 | 83 | 49 | 39 | 33 | 8.95 |
| 1 | 2 | 15 | 2 | 117 | 15 | 67 | 35 | 28 | 0 | 7.38 |
| 0 | 1 | 15 | 1 | 107 | 21 | 44 | 42 | 20 | 15 | 6.17 |
| 0 | 2 | 15 | 1 | 150 | 36 | 72 | 42 | 33 | 28 | 9.77 |
| 0 | 2 | 15 | 2 | 120 | 28 | 44 | 48 | 33 | 21 | 6.4 |
| 0 | 2 | 15 | 1 | 185 | 46 | 76 | 63 | 33 | 33 | 5.67 |
| 0 | 2 | 15 | 2 | 147 | 44 | 45 | 58 | 51 | 37 | 4.02 |
| 0 | 2 | 15 | 2 | 141 | 18 | 67 | 56 | 32 | 42 | 3.65 |
| 0 | 2 | 15 | 1 | 140 | 25 | 68 | 47 | 40 | 36 | 3.45 |
| 0 | 2 | 15 | 2 | 220 | 48 | 109 | 63 | 49 | 48 | 7.23 |
| 0 | 1 | 15 | 2 | 109 | 22 | 49 | 38 | 37 | 29 | 6.66 |
| 0 | 2 | 15 | 2 | 86 | 15 | 31 | 40 | 43 | 43 | 5.56 |
| 0 | 2 | 15 | 1 | 126 | 15 | 71 | 40 | 33 | 25 | 1.9 |
| 0 | 2 | 15 | 2 | 152 | 22 | 80 | 50 | 44 | 36 | 6.3 |
| 0 | 2 | 15 | 2 | 158 | 30 | 79 | 49 | 35 | 39 | 5.34 |
| 0 | 2 | 15 | 2 | 147 | 24 | 67 | 56 | 23 | 26 | 5.1 |
| 0 | 2 | 15 | 2 | 142 | 24 | 63 | 55 | 33 | 23 | 6.07 |
| 0 | 2 | 15 | 1 | 196 | 53 | 90 | 53 | 30 | 20 | 6.03 |
| 0 | 1 | 15 | 2 | 112 | 24 | 52 | 36 | 25 | 18 | 6.38 |
| withdraw | 2 | 15 | 2 | 149 | 26 | 77 | 46 | 27 |  | 3.56 |
| 1 | 2 | 16 | 2 | 158 | 16 | 78 | 64 | 42 | 10 | 8.07 |
| 0 | 2 | 16 | 2 | 224 | 45 | 109 | 70 | 43 | 49 | 9.37 |
| 0 | 2 | 16 | 2 | 205 | 54 | 100 | 51 | 36 | 22 | 7.82 |
| 0 | 2 | 16 | 2 | 158 | 30 | 86 | 42 | 48 | 19 | 3.29 |
| 0 | 2 | 16 | 2 | 93 | 17 | 45 | 31 | 23 | 19 | 9.02 |
| 0 | 2 | 16 | 2 | 119 | 19 | 66 | 34 | 28 | 34 | 3.94 |
| 0 | 2 | 16 | 2 | 109 | 27 | 48 | 34 | 27 | 23 | 5.64 |
| 1 | 2 | 16 | 1 | 135 | 29 | 61 | 45 | 27 | 2 | 10.57 |
| 0 | 2 | 16 | 2 | 92 | 15 | 59 | 18 | 20 | 13 | 8.63 |
| 0 | 2 | 16 | 2 | 147 | 19 | 92 | 36 | 30 | 35 | 5.57 |
| 0 | 2 | 16 | 2 | 162 | 32 | 80 | 50 | 32 | 29 | 0 |
| 0 | 2 | 16 | 2 | 128 | 23 | 71 | 34 | 31 | 20 | 11.09 |
| 1 | 1 | 16 | 2 | 186 | 41 | 92 | 53 | 23 | 3 | 4.84 |
| 0 | 1 | 16 | 2 | 138 | 29 | 64 | 45 | 34 | 22 | 0 |
| 0 | 2 | 17 | 2 | 184 | 29 | 95 | 60 | 36 | 35 | 5.13 |
| 1 | 1 | 17 | 2 | 201 | 36 | 104 | 61 | 25 | 5 | 4.08 |
| 0 | 1 | 17 | 2 | 97 | 19 | 42 | 36 | 30 | 22 | 9.13 |
| 0 | 1 | 17 | 2 | 164 | 38 | 86 | 40 | 27 | 16 | 8.05 |
| 0 | 2 | 17 | 2 | 132 | 17 | 69 | 46 | 34 | 30 | 0 |
| 0 | 2 | 17 | 2 | 185 | 39 | 85 | 61 | 36 | 23 | 9.98 |
| 0 | 2 | 17 | 2 | 142 | 25 | 60 | 57 | 34 | 32 | 0 |
| 0 | 2 | 17 | 2 | 101 | 15 | 49 | 37 | 25 | 21 | 3.3 |
| 0 | 2 | 17 | 2 | 214 | 57 | 98 | 59 | 28 | 26 | 3.08 |
