# Supplementary material for: RPS6KA5 methylation predict response to 6-week treatment for adolescent MDD patients
Source: BMC Psychiatry. 2022 Aug 19;22:561. doi: 10.1186/s12888-022-04196-4 (PMC9392312; doi:10.1186/s12888-022-04196-4)
Supplement: Supplementary file 2 — Additional file 2: Supplementary Table STab. 1. Raw data of Table 1. Demographic and clinical characteristics of MDD patients and healthy controls at baseline. [file 12888_2022_4196_MOESM2_ESM.docx]

| group(1=MDD patients,0=Controls) | sex | age | level of education | CCSQ | peer bullying | childhood abuse and neglect | adverse childhood experiences | BDI w0 | BDI w6 | RPS6KA5 methylation percentage |
| --- | --- | --- | --- | --- | --- | --- | --- | --- | --- | --- |
| 1 | 2 | 12 | 1 | 116 | 19 | 49 | 48 | 62 | 58 | 6.67 |
| 1 | 1 | 12 | 1 | 109 | 21 | 49 | 39 | 37 | 21 | 0 |
| 1 | 2 | 12 | 1 | 193 | 45 | 86 | 62 | 53 | 53 | 0 |
| 1 | 2 | 12 | 1 | 118 | 36 | 47 | 35 | 36 | 30 | 6.22 |
| 1 | 2 | 12 | 1 | 95 | 17 | 48 | 30 | 22 | 24 | 3.79 |
| 1 | 2 | 12 | 1 | 85 | 16 | 35 | 34 | 33 | 41 | 1.56 |
| 1 | 2 | 12 | 1 | 164 | 42 | 62 | 60 | 35 | 22 | 5.34 |
| 1 | 1 | 12 | 1 | 137 | 43 | 58 | 36 | 29 | withdraw | 0 |
| 1 | 1 | 13 | 1 | 131 | 28 | 58 | 45 | 30 | 35 | 8.01 |
| 1 | 2 | 13 | 1 | 156 | 15 | 108 | 33 | 40 | 28 | 8.22 |
| 1 | 2 | 13 | 1 | 165 | 52 | 71 | 42 | 37 | 35 | 7.94 |
| 1 | 2 | 13 | 1 | 87 | 24 | 30 | 33 | 33 | 36 | 5.26 |
| 1 | 2 | 13 | 1 | 102 | 19 | 53 | 30 | 34 | 31 | 6.8 |
| 1 | 2 | 13 | 1 | 194 | 15 | 121 | 58 | 60 | 53 | 9.18 |
| 1 | 2 | 13 | 1 | 157 | 42 | 47 | 68 | 32 | 44 | 0 |
| 1 | 2 | 13 | 1 | 83 | 19 | 38 | 26 | 27 | 5 | 7.35 |
| 1 | 2 | 13 | 1 | 95 | 18 | 36 | 41 | 30 | 44 | 2.87 |
| 1 | 2 | 13 | 1 | 78 | 15 | 42 | 21 | 22 | 13 | 10.92 |
| 1 | 2 | 13 | 1 | 100 | 37 | 31 | 32 | 36 | 28 | 7.88 |
| 1 | 2 | 13 | 1 | 103 | 16 | 52 | 35 | 29 | 24 | 6.4 |
| 1 | 2 | 13 | 1 | 118 | 33 | 39 | 46 | 34 | 23 | 3.49 |
| 1 | 2 | 13 | 1 | 109 | 29 | 43 | 37 | 40 | 32 | 4.02 |
| 1 | 2 | 13 | 1 | 117 | 25 | 48 | 44 | 29 | 12 | 0 |
| 1 | 2 | 13 | 1 | 76 | 18 | 36 | 22 | 20 | 39 | 4.48 |
| 1 | 2 | 13 | 1 | 74 | 18 | 29 | 27 | 30 | withdraw | 1.95 |
| 1 | 2 | 14 | 1 | 205 | 33 | 80 | 92 | 33 | 24 | 5.51 |
| 1 | 1 | 14 | 1 | 169 | 55 | 53 | 61 | 37 | 8 | 9.3 |
| 1 | 2 | 14 | 1 | 145 | 24 | 61 | 60 | 33 | 28 | 3.67 |
| 1 | 2 | 14 | 2 | 95 | 15 | 42 | 38 | 20 | 17 | 3.57 |
| 1 | 1 | 14 | 1 | 136 | 26 | 67 | 43 | 40 | 34 | 3.7 |
| 1 | 1 | 14 | 1 | 217 | 53 | 100 | 64 | 47 | 33 | 5.12 |
| 1 | 2 | 14 | 2 | 76 | 15 | 29 | 32 | 31 | 45 | 3.94 |
| 1 | 2 | 14 | 1 | 167 | 39 | 67 | 61 | 40 | 37 | 0 |
| 1 | 1 | 14 | 1 | 111 | 25 | 57 | 29 | 33 | 33 | 5.79 |
| 1 | 2 | 14 | 1 | 222 | 53 | 99 | 70 | 53 | 53 | 6.25 |
| 1 | 2 | 14 | 1 | 120 | 37 | 39 | 44 | 26 | 39 | 0 |
| 1 | 2 | 14 | 1 | 105 | 15 | 51 | 39 | 39 | 0 | 7.36 |
| 1 | 2 | 14 | 1 | 94 | 15 | 44 | 35 | 46 | 28 | 5.72 |
| 1 | 2 | 14 | 1 | 175 | 28 | 94 | 53 | 32 | 37 | 5.45 |
| 1 | 2 | 14 | 1 | 110 | 19 | 58 | 33 | 32 | 28 | 9.59 |
| 1 | 2 | 14 | 1 | 167 | 49 | 68 | 50 | 29 | 5 | 7.58 |
| 1 | 2 | 14 | 1 | 114 | 22 | 50 | 42 | 34 | 23 | 0 |
| 1 | 2 | 14 | 1 | 163 | 46 | 75 | 42 | 43 | 40 | 5.45 |
| 1 | 2 | 14 | 1 | 89 | 15 | 51 | 23 | 54 | 30 | 8.65 |
| 1 | 2 | 14 | 1 | 167 | 35 | 83 | 49 | 39 | 33 | 8.95 |
| 1 | 2 | 15 | 2 | 117 | 15 | 67 | 35 | 28 | 0 | 7.38 |
| 1 | 1 | 15 | 1 | 107 | 21 | 44 | 42 | 20 | 15 | 6.17 |
| 1 | 2 | 15 | 1 | 150 | 36 | 72 | 42 | 33 | 28 | 9.77 |
| 1 | 2 | 15 | 2 | 120 | 28 | 44 | 48 | 33 | 21 | 6.4 |
| 1 | 2 | 15 | 1 | 185 | 46 | 76 | 63 | 33 | 33 | 5.67 |
| 1 | 2 | 15 | 2 | 147 | 44 | 45 | 58 | 51 | 37 | 4.02 |
| 1 | 2 | 15 | 2 | 141 | 18 | 67 | 56 | 32 | 42 | 3.65 |
| 1 | 2 | 15 | 1 | 140 | 25 | 68 | 47 | 40 | 36 | 3.45 |
| 1 | 2 | 15 | 2 | 220 | 48 | 109 | 63 | 49 | 48 | 7.23 |
| 1 | 1 | 15 | 2 | 109 | 22 | 49 | 38 | 37 | 29 | 6.66 |
| 1 | 2 | 15 | 2 | 86 | 15 | 31 | 40 | 43 | 43 | 5.56 |
| 1 | 2 | 15 | 1 | 126 | 15 | 71 | 40 | 33 | 25 | 1.9 |
| 1 | 2 | 15 | 2 | 152 | 22 | 80 | 50 | 44 | 36 | 6.3 |
| 1 | 2 | 15 | 2 | 158 | 30 | 79 | 49 | 35 | 39 | 5.34 |
| 1 | 2 | 15 | 2 | 147 | 24 | 67 | 56 | 23 | 26 | 5.1 |
| 1 | 2 | 15 | 2 | 142 | 24 | 63 | 55 | 33 | 23 | 6.07 |
| 1 | 2 | 15 | 1 | 196 | 53 | 90 | 53 | 30 | 20 | 6.03 |
| 1 | 1 | 15 | 2 | 112 | 24 | 52 | 36 | 25 | 18 | 6.38 |
| 1 | 2 | 15 | 2 | 149 | 26 | 77 | 46 | 27 | withdraw | 3.56 |
| 1 | 2 | 16 | 2 | 158 | 16 | 78 | 64 | 42 | 10 | 8.07 |
| 1 | 2 | 16 | 2 | 224 | 45 | 109 | 70 | 43 | 49 | 9.37 |
| 1 | 2 | 16 | 2 | 205 | 54 | 100 | 51 | 36 | 22 | 7.82 |
| 1 | 2 | 16 | 2 | 158 | 30 | 86 | 42 | 48 | 19 | 3.29 |
| 1 | 2 | 16 | 2 | 93 | 17 | 45 | 31 | 23 | 19 | 9.02 |
| 1 | 2 | 16 | 2 | 119 | 19 | 66 | 34 | 28 | 34 | 3.94 |
| 1 | 2 | 16 | 2 | 109 | 27 | 48 | 34 | 27 | 23 | 5.64 |
| 1 | 2 | 16 | 1 | 135 | 29 | 61 | 45 | 27 | 2 | 10.57 |
| 1 | 2 | 16 | 2 | 92 | 15 | 59 | 18 | 20 | 13 | 8.63 |
| 1 | 2 | 16 | 2 | 147 | 19 | 92 | 36 | 30 | 35 | 5.57 |
| 1 | 2 | 16 | 2 | 162 | 32 | 80 | 50 | 32 | 29 | 0 |
| 1 | 2 | 16 | 2 | 128 | 23 | 71 | 34 | 31 | 20 | 11.09 |
| 1 | 1 | 16 | 2 | 186 | 41 | 92 | 53 | 23 | 3 | 4.84 |
| 1 | 1 | 16 | 2 | 138 | 29 | 64 | 45 | 34 | 22 | 0 |
| 1 | 2 | 17 | 2 | 184 | 29 | 95 | 60 | 36 | 35 | 5.13 |
| 1 | 1 | 17 | 2 | 201 | 36 | 104 | 61 | 25 | 5 | 4.08 |
| 1 | 1 | 17 | 2 | 97 | 19 | 42 | 36 | 30 | 22 | 9.13 |
| 1 | 1 | 17 | 2 | 164 | 38 | 86 | 40 | 27 | 16 | 8.05 |
| 1 | 2 | 17 | 2 | 132 | 17 | 69 | 46 | 34 | 30 | 0 |
| 1 | 2 | 17 | 2 | 185 | 39 | 85 | 61 | 36 | 23 | 9.98 |
| 1 | 2 | 17 | 2 | 142 | 25 | 60 | 57 | 34 | 32 | 0 |
| 1 | 2 | 17 | 2 | 101 | 15 | 49 | 37 | 25 | 21 | 3.3 |
| 1 | 2 | 17 | 2 | 214 | 57 | 98 | 59 | 28 | 26 | 3.08 |
| 0 | 2 | 12 | 2 | 62 | 15 | 29 | 18 | 2 |  |  |
| 0 | 2 | 12 | 2 | 61 | 15 | 28 | 18 | 0 |  |  |
| 0 | 2 | 12 | 1 | 80 | 15 | 35 | 30 | 0 |  |  |
| 0 | 2 | 12 | 1 | 123 | 21 | 49 | 53 | 4 |  |  |
| 0 | 2 | 12 | 1 | 104 | 23 | 50 | 31 | 13 |  |  |
| 0 | 2 | 13 | 1 | 108 | 17 | 62 | 29 | 4 |  |  |
| 0 | 2 | 13 | 1 | 102 | 29 | 47 | 26 | 19 |  |  |
| 0 | 2 | 13 | 1 | 75 | 20 | 32 | 23 | 8 |  |  |
| 0 | 2 | 13 | 1 | 65 | 15 | 31 | 19 | 2 |  |  |
| 0 | 2 | 13 | 1 | 77 | 22 | 30 | 25 | 1 |  |  |
| 0 | 2 | 13 | 1 | 54 | 15 | 29 | 10 | 0 |  |  |
| 0 | 2 | 13 | 1 | 112 | 19 | 64 | 29 | 1 |  |  |
| 0 | 2 | 13 | 1 | 72 | 18 | 34 | 20 | 13 |  |  |
| 0 | 2 | 13 | 2 | 70 | 17 | 29 | 24 | 8 |  |  |
| 0 | 2 | 13 | 2 | 74 | 16 | 29 | 29 | 11 |  |  |
| 0 | 2 | 13 | 1 | 83 | 15 | 42 | 26 | 14 |  |  |
| 0 | 2 | 13 | 1 | 94 | 15 | 52 | 27 | 10 |  |  |
| 0 | 2 | 13 | 1 | 73 | 22 | 34 | 17 | 8 |  |  |
| 0 | 2 | 13 | 1 | 71 | 19 | 29 | 23 | 13 |  |  |
| 0 | 1 | 13 | 2 | 60 | 15 | 29 | 16 | 0 |  |  |
| 0 | 2 | 13 | 2 | 60 | 15 | 29 | 16 | 0 |  |  |
| 0 | 2 | 14 | 1 | 128 | 24 | 64 | 40 | 3 |  |  |
| 0 | 1 | 14 | 1 | 60 | 15 | 29 | 16 | 4 |  |  |
| 0 | 2 | 14 | 1 | 108 | 25 | 57 | 26 | 15 |  |  |
| 0 | 2 | 14 | 2 | 72 | 16 | 33 | 23 | 4 |  |  |
| 0 | 1 | 14 | 1 | 83 | 17 | 40 | 26 | 15 |  |  |
| 0 | 1 | 14 | 1 | 73 | 22 | 34 | 17 | 8 |  |  |
| 0 | 1 | 15 | 1 | 60 | 15 | 29 | 16 | 0 |  |  |
| 0 | 1 | 15 | 1 | 92 | 15 | 52 | 25 | 10 |  |  |
| 0 | 2 | 15 | 1 | 67 | 19 | 29 | 19 | 13 |  |  |
| 0 | 1 | 15 | 1 | 88 | 27 | 34 | 27 | 6 |  |  |
| 0 | 2 | 15 | 1 | 63 | 16 | 30 | 17 | 1 |  |  |
| 0 | 2 | 15 | 1 | 104 | 25 | 57 | 22 | 15 |  |  |
| 0 | 2 | 15 | 1 | 104 | 25 | 57 | 22 | 15 |  |  |
| 0 | 2 | 15 | 1 | 83 | 17 | 40 | 26 | 15 |  |  |
| 0 | 2 | 15 | 1 | 68 | 16 | 31 | 21 | 0 |  |  |
| 0 | 2 | 15 | 1 | 60 | 15 | 29 | 16 | 0 |  |  |
| 0 | 1 | 15 | 1 | 92 | 39 | 29 | 24 | 3 |  |  |
| 0 | 1 | 15 | 1 | 76 | 20 | 33 | 23 | 3 |  |  |
| 0 | 2 | 15 | 1 | 84 | 18 | 41 | 25 | 9 |  |  |
| 0 | 2 | 15 | 1 | 67 | 16 | 30 | 21 | 0 |  |  |
| 0 | 2 | 15 | 1 | 140 | 25 | 86 | 29 | 7 |  |  |
| 0 | 2 | 15 | 2 | 114 | 33 | 51 | 30 | 6 |  |  |
| 0 | 2 | 15 | 1 | 75 | 15 | 42 | 18 | 14 |  |  |
| 0 | 2 | 16 | 1 | 112 | 16 | 72 | 24 | 27 |  |  |
| 0 | 2 | 16 | 2 | 60 | 15 | 29 | 16 | 0 |  |  |
| 0 | 1 | 16 | 2 | 60 | 15 | 29 | 16 | 0 |  |  |
| 0 | 2 | 16 | 1 | 112 | 24 | 64 | 24 | 3 |  |  |
| 0 | 1 | 16 | 1 | 60 | 15 | 29 | 16 | 4 |  |  |
| 0 | 1 | 16 | 2 | 72 | 16 | 33 | 23 | 4 |  |  |
| 0 | 2 | 16 | 2 | 66 | 16 | 29 | 21 | 11 |  |  |
| 0 | 1 | 17 | 2 | 151 | 47 | 70 | 34 | 24 |  |  |
| 0 | 1 | 17 | 2 | 66 | 17 | 29 | 20 | 8 |  |  |
